# Environmental Carcinogens and Cancer Risk

**DOI:** 10.3390/cancers13040622

**Published:** 2021-02-04

**Authors:** Nicole M. Gatto

**Affiliations:** School of Community and Global Health, Claremont Graduate University, 150 E. 10th Street, Claremont, CA 91711, USA; nicole.gatto@cgu.edu

The year 2022 will mark the 60th anniversary of the 1962 publication of Rachel Carson’s seminar work *Silent Spring* [1], which questioned the judiciousness of widespread use of chemical pesticides in the environment, initiating the modern environmental movement that still endures today. In the United States, the 1970s saw the first Earth Day and the passage of many of our important environmental protection laws, including the Clean Air Act, the Clean Water Act, and the Toxic Substances Control Act, as well as the establishment of federal agencies with responsibilities for environmental health, namely the Environmental Protection Agency (EPA) and the Occupational Health and Safety Administration (OSHA). The significance of the potential impacts of environmental sources, including polluted air and water, hazardous waste, and unsafe workplaces, on human health was recognized through these national laws, along with the responsibilities delegated to corresponding regulatory agencies [2]. 

About one-fifth of people worldwide and one-third of people in industrialized countries will be diagnosed with cancer during their lifetimes [3]. Environmental carcinogens encompass a broad range of chemical, biological, and physical agents, as well as some lifestyle factors. Despite small relative risks of cancer from exposure to environmental carcinogens, attributable risks have the potential to be large because of the high prevalence of exposure [4]. The World Health Organization (WHO) estimates that 20% of cancers worldwide are attributable to environmental risks, predominantly air pollution, management of chemicals, radiation, and workers’ protection [5]. The cancer burden from environmental agents varies by the degree of industrialization of the country [6]. A unifying characteristic of these agents is the involuntary nature of their exposure from environmental and occupational settings. 

The EPA’s Integrated Risk Information System (IRIS) program created in 1985 identifies and characterizes human health hazards associated with chemicals found in the environment. To date, the IRIS’s weight-of-evidence approach has identified over 150 of such chemicals as carcinogenic, likely to be carcinogenic, or suggestive evidence of carcinogenic potential [7]. The International Agency for Research on Cancer (IARC) of the World Health Organization (WHO) through its monographs also evaluates potential environmental carcinogens. Since 1971, the IARC has evaluated more than 1000 agents through the monographs, and more than 500 have been identified as carcinogenic, probably carcinogenic, or possibly carcinogenic to humans [8]. Necessary for both these syntheses are primary studies using humans and experimental models. 

Today, the legacy of the historical actions from past decades is threatened. The growing use of environmental chemicals, the changing climate, and the waxing and waning of support for environmental protections by political administrations all jeopardize the progress we have achieved. Simply considering the tens of thousands of chemicals in use across the world, substantial work remains to understand the potential cancer risk to humans as well as the biological mechanisms of action. Therefore, the need for research is greater than ever.
